# Protein-Protein Interactions: Insight from Molecular Dynamics Simulations and Nanoparticle Tracking Analysis

**DOI:** 10.3390/molecules26185696

**Published:** 2021-09-20

**Authors:** Wei Lim Chong, Koollawat Chupradit, Sek Peng Chin, Mai Mai Khoo, Sook Mei Khor, Chatchai Tayapiwatana, Piyarat Nimmanpipug, Weeraya Thongkum, Vannajan Sanghiran Lee

**Affiliations:** 1 Department of Chemistry, Faculty of Science, Universiti Malaya, Kuala Lumpur 50603, Malaysia; wlchongwilliam@gmail.com (W.L.C.); maimaikhoo1411@siswa.um.edu.my (M.M.K.); naomikhor@um.edu.my (S.M.K.); 2 Division of Clinical Immunology, Department of Medical Technology, Faculty of Associated Medical Sciences, Chiang Mai University, Chiang Mai 50200, Thailand; kool_krub@msn.com (K.C.); asimi002@hotmail.com (C.T.); 3 Center of Biomolecular Therapy and Diagnostic, Faculty of Associated Medical Sciences, Chiang Mai University, Chiang Mai 50200, Thailand; weeraya.t@cmu.ac.th; 4 Department of Pharmaceutical Chemistry, Faculty of Pharmacy, Universiti Malaya, Kuala Lumpur 50603, Malaysia; spchin@um.edu.my; 5 Department of Chemistry, Faculty of Science, Chiang Mai University, Chiang Mai 50200, Thailand; piyarat.n@cmu.ac.th; 6 Center of Excellence for Innovation in Analytical Science and Technology (I-ANALY-S-T), Chiang Mai University, Chiang Mai 50200, Thailand; 7 Center of Innovative Immunodiagnostic Development, Department of Medical Technology, Faculty of Associated Medical Sciences, Chiang Mai University, Chiang Mai 50200, Thailand

**Keywords:** nanoparticle tracking analysis, molecular dynamics simulations, HIV-1, DARPins, protein-protein binding

## Abstract

Protein-protein interaction plays an essential role in almost all cellular processes and biological functions. Coupling molecular dynamics (MD) simulations and nanoparticle tracking analysis (NTA) assay offered a simple, rapid, and direct approach in monitoring the protein-protein binding process and predicting the binding affinity. Our case study of designed ankyrin repeats proteins (DARPins)—AnkGAG1D4 and the single point mutated AnkGAG1D4-Y56A for HIV-1 capsid protein (CA) were investigated. As reported, AnkGAG1D4 bound with CA for inhibitory activity; however, it lost its inhibitory strength when tyrosine at residue 56 AnkGAG1D4, the most key residue was replaced by alanine (AnkGAG1D4-Y56A). Through NTA, the binding of DARPins and CA was measured by monitoring the increment of the hydrodynamic radius of the AnkGAG1D4-gold conjugated nanoparticles (AnkGAG1D4-GNP) and AnkGAG1D4-Y56A-GNP upon interaction with CA in buffer solution. The size of the AnkGAG1D4-GNP increased when it interacted with CA but not AnkGAG1D4-Y56A-GNP. In addition, a much higher binding free energy (∆GB) of AnkGAG1D4-Y56A (−31 kcal/mol) obtained from MD further suggested affinity for CA completely reduced compared to AnkGAG1D4 (−60 kcal/mol). The possible mechanism of the protein-protein binding was explored in detail by decomposing the binding free energy for crucial residues identification and hydrogen bond analysis.

## 1. Introduction

In almost all significant cellular processes and biological functions, protein-protein interactions play a vital role [1]. Identifying protein-protein interactions and their binding affinity are crucial in knowing cellular biological processes, discovery and design of novel therapeutics, protein engineering, and mutagenesis studies [2]. Hence, convenient, highly sensitive, and low-cost bioanalytical tools that allow fast and high throughput screening targeting protein-protein interactions are extremely important for biomolecular research [3,4,5].

Various computational tools are available for the evaluation of the functional relevance of the predicted protein-protein complexes and the prediction of their realistic binding affinity [6]. Molecular dynamics (MD) simulation is one of the main in silico tool in the study of biomolecules owing to its better predictive power and more reliable analysis of protein structure, dynamics, and functions [7,8]. In addition, simulating protein-protein interactions in the presence of water through molecular dynamics (MD) simulations has become a common, accurate and reliable approach in understanding the communication between the proteins. Dynamics and structure of protein obtained from MD simulations can be analyzed in depth to understand the interactions of the protein with its targets [7] and reveal the amino acids that are critical to enabling the communication [9,10] or the other way, disrupting the interactions [11]. Furthermore, predicted binding free energy calculated from MD snapshots can distinguish the candidate amino acids sequences [12,13,14,15,16,17] for their affinity in binding with a target.

Nevertheless, investigating the protein-protein binding interactions using the experimental technique is indispensable. Nanoparticle tracking analysis (NTA) emerges as an innovative and attractive approach commonly employed in routine to visualize and analyze nanoparticles in liquids directly, in real time since it was first introduced in 2006 [18]. Each of the particles scatters light when they are illuminated by a laser beam and the paths taken by the particles under Brownian motion over a period of time is recorded using a camera [19]. Hydrodynamic radius (r_h_), or the size of each tracked individual particle can be measured based on the displacement of the particle over time [20,21,22] using the Stokes-Einstein equation, where K_B_ is Boltzmann’s constant, T is temperature, and η is viscosity.


(1) $$\mathrm{Dt}=\frac{K_{B}T}{6{\pi \eta r}_{h}}$$

In numerous studies, nanoparticle tracking analysis (NTA) has demonstrated its strength in the direct and quantitative measurement of the protein-protein binding [23,24,25,26,27,28] by coupling gold nanoparticle probes and dynamic light scattering (DLS) as a light scattering enhancer and read-out system, respectively. Of the highlights, NTA was not only successful in confirming the completion of conjugation between a protein and gold nanoparticles, but also detected and monitored the binding of antigen toward the antibody-gold conjugated in situ by measuring the average particle size change of the assay solution [26].

In this work, we have adopted MD simulations and NTA technique as a novel protocol to decipher the binding patterns and monitor the binding between the Designed Ankyrin Repeat Proteins (DARPins) Ank^GAG^1D4/Ank^GAG^1D4-Y56A and HIV-1 capsid protein (CA). DARPins are genetically engineered antibody mimetic proteins derived from naturally occurring ankyrin proteins, typically exhibiting highly specific and high-affinity target protein binding in a wide variety of bacterial and mammalian cells [29,30,31,32]. DARPins are composed of stacked repeats containing 33 amino acids. Each repeat is formed by two antiparallel *α*-helices and a *β*-turn connecting the next repeat [33,34]. These repeats are flanked by constant capping regions, forming one contiguous polypeptide chain. Ank^GAG^1D4 is a trimodular DARPin binding to HIV-1 capsid protein (CA) and was isolated from screening a phage-display artificial library [35]. Ank^GAG^1D4 exerted its intracellular antiviral activity at the late phase of the HIV-1 life cycle, by negatively interfering with the Gag protein assembly and budding machinery [35]. To increase the affinity of Ank^GAG^1D4 in binding with CA, a few amino acid residues located in the interacting site were subjected to site-directed mutagenesis [36] and binding affinity of Ank^GAG^1D4 for CA was lost when a mutation occurs at residue Y56 of Ank^GAG^1D4 (Ank^GAG^1D4-Y56A). Through all atoms’ MD simulations, we were able to visualize the conformational changes of the CA upon the binding of Ank^GAG^1D4 and Ank^GAG^1D4-Y56A and explain the reason for the unfavorable binding of the mutant DARPins toward CA. The capability of NTA in monitoring the binding process between two proteins was found useful to examine if/whether binding occurs between derivatives of Ank^GAG^1D4 and CA. Moreover, structural, and dynamic properties obtained from MD simulations permit exploration of possible mechanisms that led to different binding affinities of DARPins.

## 2. Results

### 2.1. Single Point Mutation Disrupts the Interaction Network between Ank^GAG^1D4 and HIV-1 CA 

Binding free energy, ∆_GB_ computed under MMGBSA and ∆_PB_ obtained from MMPBSA protocols have differentiated the affinity of Ank^GAG^1D4 and Ank^GAG^1D4-Y56A for CA as described in Table 1. Ank^GAG^1D4 has ∆_GB_ of −60.38 kcal/mol while Ank^GAG^1D4-Y56A has ∆_GB_ of −31.06 kcal/mol. The magnitude of the energy difference between the two complexes obtained from MMGBSA calculations is more pronounced as ∆_PB_ of Ank^GAG^1D4 and Ank^GAG^1D4-Y56A in complex with CA was −77.49 kcal/mol and −67.07 kcal/mol, respectively. Binding free energy accounted from both methods agree with the experimental findings [36] in which Ank^GAG^1D4 lost its affinity toward CA when tyrosine (Y) at position 56 was mutated to alanine (A). In both the complexes, favorable contributions to the binding arose from van der Waals (vdW) and the non-polar part of the solvation free energy, as opposed to the unfavorable total electrostatic contributions (EEL + E_GB_ and EEL + E_pb_). Site-directed mutagenesis at the residue Y56 has disrupted all the energy components involved in the binding, where the vdW interactions lost are observed to be greater (Table 1).

**Figure 1:**
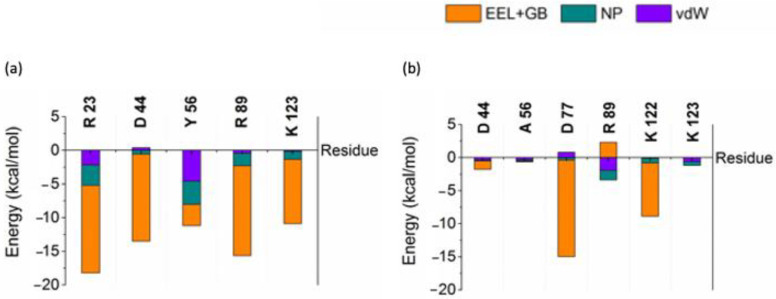
Interaction free energy of key residues (**a**) Ank^GAG^1D4 and (**b**) Ank^GAG^1D4-Y56A in complex.

Interaction free energy (IE) of residues was calculated by decomposing the binding free energy into vdW, non-polar contributions to the solvation free energy (NP) and the sum of electrostatic interactions (EEL) and electrostatic contribution to the solvation free energy components (GB) to identify the important residues contributing for the binding affinity using residues within close contacts (4 Å) between Ank^GAG^1D4/Ank^GAG^1D4-Y56 and CA. Key residues R23, D44, Y56, R89, and K123 of Ank^GAG^1D4 shown in Figure 1 were previously identified [37] for their crucial role in binding affinity observed to have remarkable low IE with major contribution from the sum of EEL and GB. Interactions of D44, Y56A, R89 and K123 with CA reduced drastically as IE went higher in Ank^GAG^1D4-Y56-CA. IE Y56A increased the most among the key residues, about 95%, from −11.17 kcal/mol to −0.65 kcal/mol. 

**Figure 2:**
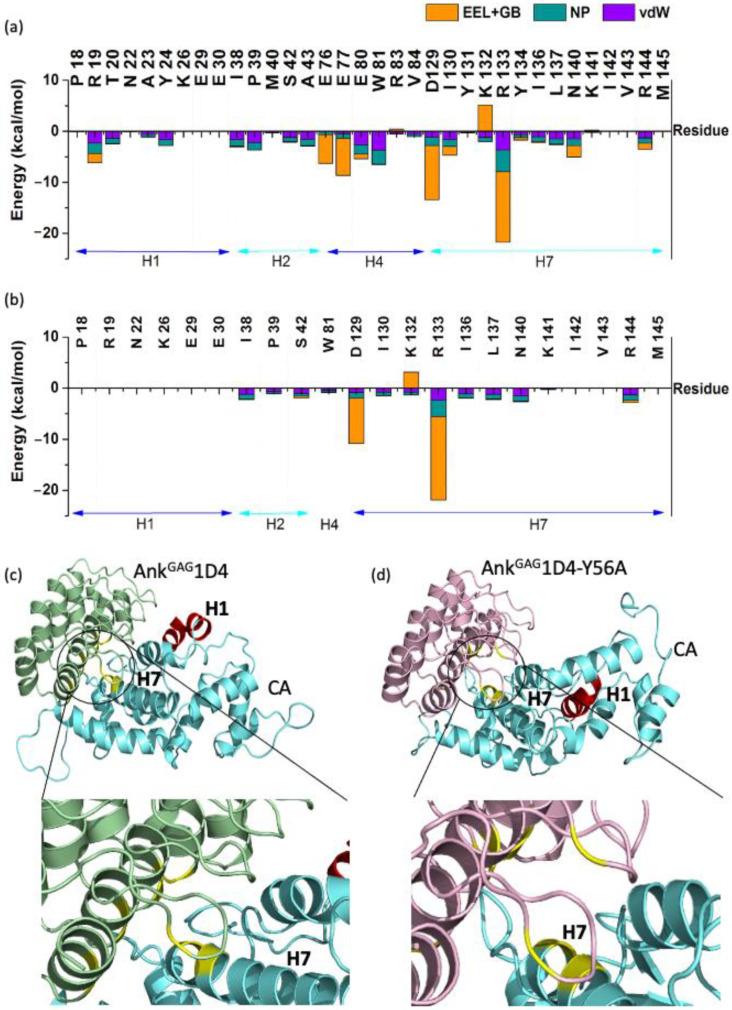
Interaction free energy of the CA residues interacting with (**a**) Ank^GAG^1D4 and (**b**) Ank^GAG^1D4-Y56A within 4 Å binding vicinity. Comparison between the conformation of (**c**) Ank^GAG^1D4-CA and (**d**) Ank^GAG^1D4-Y56-CA complexes after 100 ns simulations showed that the H1 (red) moved further away from the DARPin in (**d**). Residues in both complexes that contributed the most in the binding free energy were highlighted in yellow.

In addition, different interaction patterns of CA helices in complexed with Ank^GAG^1D4 and Ank^GAG^1D4-Y56 was observed as illustrated in Figure 2. Residues of helix 7 (H7), helix 4 (H4), helix 1 (H1), and helix 2 (H2) were in close contact with the Ank^GAG^1D4 with a total IE of −52.32 kcal/mol, −28.08 kcal/mol, −12.82 kcal/mol, and −12.13 kcal/mol, respectively. Among the close contact residues of CA, E46 located in the loop between H2 and H3, D129 and R133 of H7 are the major binding affinity contributors by making favorable interaction with Ank^GAG^1D4 via R89, D44, and K123 with IE of −17.05 kcal/mol (E46-R89), −12.31 kcal/mol (R133-D44), and −9.96 kcal/mol (D129-K123). In contrast, interactions between Ank^GAG^1D4-Y56A and helices of CA disrupted as H1 and H2 CA did not interact with Ank^GAG^1D4-Y56A. Total IE of H7 residues in Ank^GAG^1D4-Y56A-CA was −42.46 kcal/mol, about 10 kcal/mol or 18% higher than that of H7 in Ank^GAG^1D4-CA. Apart from energetic profile, single point mutation Y56A has dynamically changed the conformation of the CA as H1 in Ank^GAG^1D4-Y56-CA was observed to have moved further away from the DARPin (Figure 2). 

**Figure 3:**
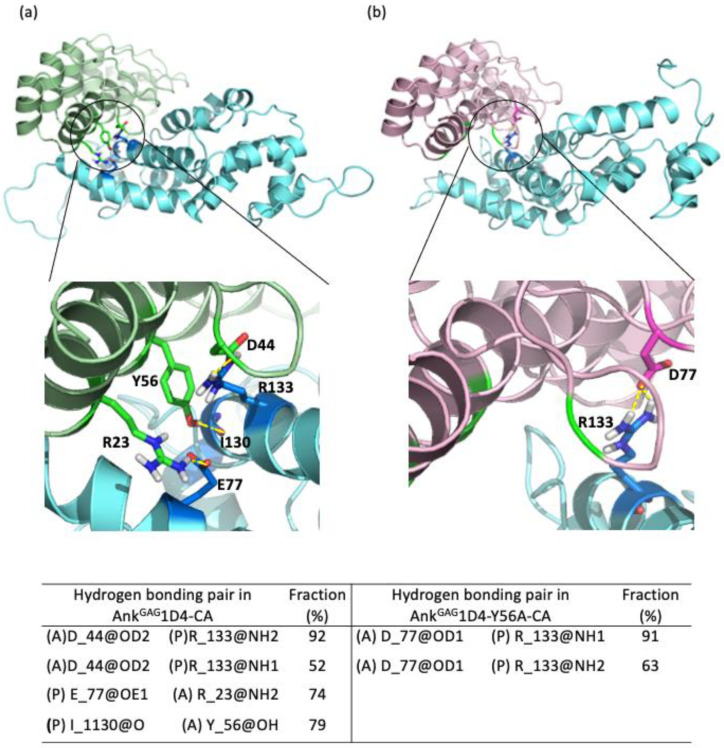
Important hydrogen bonding pairs of both the (**a**) Ank^GAG^1D4-CA and (**b**) Ank^GAG^1D4-Y56A-CA complexes. R23, D44, and Y56 (green sticks) of Ank^GAG^1D4 making hydrogen bonds with CA (blue sticks) while the hydrogen bond between D77 and R133 in Ank^GAG^1D4-Y56A-CA is shown in pink and blue sticks. R23, D44, and Y56 that are making hydrogen bonds with the CA in Ank^GAG^1D4-CA are at a distance that is too far to establish hydrogen bonds at the Ank^GAG^1D4-Y56A-CA complex (green shading in **b**). The table lists the hydrogen lifetime where (A) denotes residues from Ank^GAG^1D4-CA/Ank^GAG^1D4-Y56A while (P) denotes residues from CA.

Hydrogen bonding interactions play a role in stabilizing the inter-molecular contacts. Hydrogen bond analyses on the MD trajectories were carried out and their fractions, which reflect the percentage of conservation, were reported in Figure 3. A total of 12 hydrogen bonds that remained more than 50% of the simulations time are found in the Ank^GAG^1D4-CA complex compared to 11 in the Ank^GAG^1D4-Y56A-CA complex. It is noticeable that more than half of the hydrogen bonding pairs involved water molecules at the binding vicinity (Table S1, Supplementary Materials). Presence of tyrosine (Y) at position 56 in Ank^GAG^1D4 promoted hydrogen bond formation with residue I130 of CA. The hydrogen bonding pairs between CA and Ank^GAG^1D4 at R133-D44, E77-R23, and I130-Y56 were absent upon the substitution of tyrosine with alanine at position 56. In the Ank^GAG^1D4-Y56A-CA complex, a new hydrogen bonding pair was found between R133-D77. Y56A mutation reorganized the hydrogen bonding network within the complex (Figure 3) and with surrounding water molecules. This agrees with the free energy binding analysis, in which the affinity was reduced in the Ank^GAG^1D4-Y56A-CA complex.

### 2.2. Confirming Binding of Ank^GAG^1D4 toward CA

#### 2.2.1. NTA Assay

**Figure 4:**
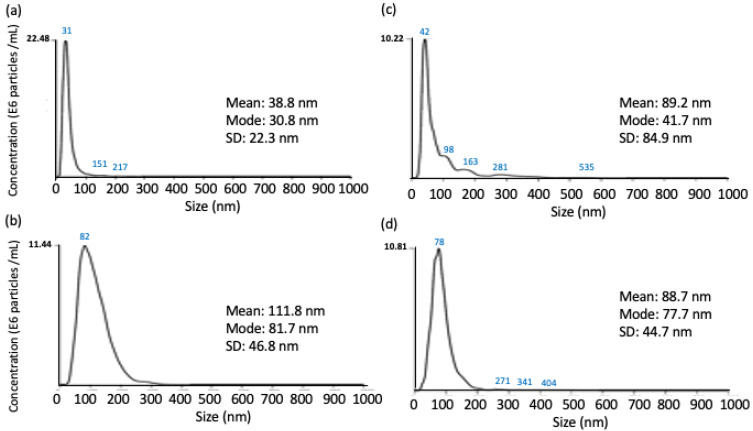
The average size of DARPin-conjugated colloidal gold before and after binding with HIV-1 CA. Size of (**a**) Ank^GAG^1D4-conjugated colloidal gold, (**b**) Ank^GAG^1D4-Y56A conjugated colloidal gold, (**c**) Ank^GAG^1D4 conjugated colloidal gold upon binding with CA and (**d**) Ank^GAG^1D4-Y56A conjugated colloidal gold upon binding with CA under measurement of nanoparticle tracking analysis.

NTA-DLS technique would be an alternative approach for directly and quantitatively measuring the effect of point mutation on the binding affinity between a protein-conjugated gold and a target analyte in solution. Upon protein binding, the size of the protein particles in a solution containing two binding proteins would increase, equivalent to the summation of the size of the two binding proteins. The average size of Ank^GAG^1D4-conjugated GNP measured by NTA was 31 nm (Figure 4a) and was much smaller than Ank^GAG^1D4-Y56A-conjugated GNP that was 82 nm (Figure 4b). This may be due to the preparation step of the Ank conjugated gold particles that caused variations in particle size distribution. Interestingly, after adding the HIV-1 CA, the size of Ank^GAG^1D4-conjugated GNP significantly increased from 31 nm to 42 nm and some larger particles with sizes 98 nm, 163 nm and 281 nm were found forming in the solution (Figure 4c). However, the size of the Ank^GAG^1D4-Y56A-conjugated GNP decreased slightly to 78 nm (Figure 4d). The size of Ank^GAG^1D4-conjugate GNP almost remained unchanged suggested that Ank^GAG^1D4-Y56A did not bind with CA while an increase in the size of Ank^GAG^1D4-conjugated GNP showed that the binding occurs between Ank^GAG^1D4 and CA. Additionally, the larger size of Ank^GAG^1D4-conjugate GNP after binding with CA affected the Brownian movements of the particles. Observation obtained from NTA was therefore consistent with the previous study [36]. Protein binding can be distinguished from protein aggregation by monitoring the size of the protein particles after the binding reaction.

#### 2.2.2. Electrochemical Impedance Spectroscopy

**Figure 5:**
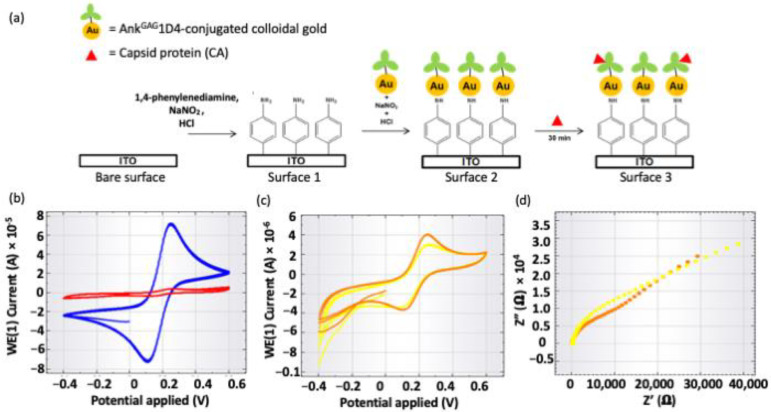
Simple stepwise of (**a**) fabrication of ITO surface used for detecting binding of CA, and the cyclic voltammograms of the (**b**) bare ITO surface (blue) and Surface 1 (red), (**c**) Surface 2 (orange) and Surface 3 (yellow), and (**d**) the Nyquist plots obtained from the EIS measurements for Surface 2 (orange) and Surface 3 (yellow).

Cyclic voltammetry (CV) was used to investigate the electrochemical properties of the fabricated ITO surface using [Fe(CN)_6_]^4−/3−^ redox active species after every single step of ITO surface fabrication (Figure 5). Figure 5b shows the cyclic voltammograms of bare ITO surface and 1,4-phenylenediamine-fabricated ITO surface (Surface 1), whereas Figure 5c shows the CV of Surface 2 and Surface 3, respectively. A pair of well-defined Faradaic peaks for [Fe(CN)_6_]^4−/3−^ species was found on the reversible CV of bare ITO surface (blue color curve in Figure 5b). It was due to the absence of the inhibition layer on the ITO surface, hence the redox species could directly access the ITO surface. After the electrochemical deposition of 1,4-phenylenediamine, no Faradaic peaks for redox species were observed between +0.6 V and −0.4 V. Deposition of 1,4-phenylenediamine inhibited the redox species from accessing the ITO working electrode. The attachment of Ank^GAG^1D4 conjugated colloidal gold to 1,4-phenylenediamine on the ITO surface showed no significant changes in peak current. This was because the high conductivity of gold increased peak current, whereas the large molecular size of Ank^GAG^1D4 could cause the peak current to decrease, therefore the change in peak current had been balanced out by these two factors. The decrease of peak currents (4 µA to 3 µA) in Figure 5c and the increase of charge transfer resistivity (18.511 kΩ to 31.791 kΩ) obtained by fitting the Nyquist plots with the equivalent circuit in the inset of Figure 5d showing that the binding of large CA to the surface-bound Ank^GAG^1D4 decreased the access of redox species to the ITO working electrode. Therefore, the changes in electrochemical signal (e.g., peak current and charge transfer resistivity) indicated binding of Ank^GAG^1D4 and CA was detected and hence validated the binding activity. 

## 3. Discussion

Binding free energy computed by MMGBSA or MMPBSA algorithms have been widely employed to predict the binding affinity of protein-ligand [38,39,40,41] and protein-protein complexes [16,42,43,44] and is in good agreement with experimental findings. In this work, binding free energy of Ank^GAG^1D4 and Ank^GAG^1D4-Y56A for HIV-1 CA was computed to understand the effect of single point mutation performed on residue Y56 of Ank^GAG^1D4. Residue Y56 was previously identified as one of the crucial residues of Ank^GAG^1D4 for CA binding affinity. Binding free energy (Δ_GB_) of Ank^GAG^1D4-Y56A-CA (−31.06 kcal/mol) increased almost two folds as compared to Ank^GAG^1D4-CA (−60.38 kcal/mol). The effect of mutating just one amino acid of DARPin could be huge and significant as single point mutation could improve the binding affinity of DARPin for its target [44,45] but it could also cause DARPin to lose its affinity for its target entirely such as Ank^GAG^1D4. Substituting tyrosine with alanine at residue 56 Ank^GAG^1D4 had altered the binding interactions between Ank^GAG^1D4 and CA as favorable inter-molecular contacts reduced within the complex. There were nine CA residues, R19, E76, E77, E80, W81, D129, I130, R133 and N140 from H1, H4 and H7 interacted with Ank^GAG^1D4 with IE < −5 kcal/mol while only two CA residues D129 and R133 were low in IE upon interactions with Ank^GAG^1D4-Y56A. In the Ank^GAG^1D4-Y56A-CA complex, H1 of CA and R23, D44 and Y56 of Ank^GAG^1D4-Y56A have shifted away from the binding interface. Consequently, hydrogen bonds were not formed between R23, D44 and Y56 and CA in the Ank^GAG^1D4-Y56A-CA complex as their distance is too far for hydrogen bonding. Hence, conformational changes and hydrogen bonding network reorganization making the protein-protein interactions unfavorable between Ank^GAG^1D4-Y56A and CA. Conventional approaches in investigating protein-protein binding are normally tedious and require high cost and long incubation time. NTA assay comes into place to simplify the process and offering a rapid and simple technique to investigate and monitor the binding between two proteins in solution [26]. The binding process was determined and monitored by measuring the hydrodynamic radius of the gold conjugated nanoparticles (GNP) of Ank^GAG^1D4 and Ank^GAG^1D4-Y56A after mixing with CA in solution. In the mixed solution, Ank^GAG^1D4-conjugated GNP has its size increased from 31 nm to 42 nm while the size of Ank^GAG^1D4-Y56A-conjugated GNP almost remained unchanged. The size increment of Ank^GAG^1D4-conjugated GNP inferred that binding occurred between Ank^GAG^1D4 and CA. Size of Ank^GAG^1D4-Y56A-conjugated GNP did not increase suggested no binding occurred between the two proteins. It was in agreement with previous ELISA findings where Ank^GAG^1D4-Y56A did not bind with CA [36]. Due to a larger size, Brownian movements of Ank^GAG^1D4 was observed to reduce after binding with CA. Apart from MD simulations and NTA, binding activity was also validated by the EIS method. EIS offers a highly sensitive and selective technique for most bio-sensor studies involving large biomolecules, such as proteins [45,46,47]. The detection principle works on measuring changes of electrochemical signals such as charge transfer [48,49], capacitance [50] or impedance [51,52]. Binding between Ank^GAG^1D4 and CA was detected as changes in electrochemical signals such as peak current and charge transfer resistivity were observed (Figure 5). The large size of CA prevented redox species to interact with the ITO working electrode resulted in changes in the electrochemical signals. For the first time, our work showcasing how MD simulations and NTA techniques can be applied to provide mechanistic insights into protein-protein interactions and protein-protein association. The techniques described are practical and simple in elucidating the impacts of a single point mutation toward the protein-protein binding, including the binding and dynamics pattern change. Additionally, it would allow predicted binding interactions between computationally designed proteins and target to be easily verified.

## 4. Materials and Methods

### 4.1. Protein-Protein Docking 

X-ray crystal structures of Ank^GAG^1D4 and Ank^GAG^1D4-Y56A were retrieved from the Protein Data Bank (PDB) with PDB ID 4HLL and 4ZFH, respectively. Ank^GAG^1D4 was docked to the helix 7 of CA under rigid-body docking protocol embedded in Z-Dock Discovery Studio programme 2.5 as described in the previous study [37]. The complex of Ank^GAG^1D4-Y56A-CA was then generated by superimposing the Ank^GAG^1D4-Y56A to Ank^GAG^1D4 in the Ank^GAG^1D4-CA complex. Both complexes underwent minimization under RMS gradient tolerance of 0.1000 kcal / (mol × Angstrom). CHARMm force field with Momany-Rone partial charge [53] was applied to describe the molecular properties of the protein structures.

### 4.2. Molecular Dynamics 

Both minimized Ank^GAG^1D4-CA and Ank^GAG^1D4-Y56A-CA complexes were subjected to propka [54] to assign the protonation state of the amino acid residues in the complexes. Then, the two structures were prepared under the LeaP module embedded in the AMBER 14 program [55], for adding the missing hydrogen and solvating the complex using a TIP3P water box with counter ions added to neutralize the system. FF14SB force field [56] has been used to describe the protein complexes. MD simulations were performed using PMEMD.CUDA from AMBER with a time step of 2 fs and a cutoff radius of 10 Å for the nonbonded interactions, and particle-mesh Ewald (PME) was used for calculating the long-range electrostatic interactions. SHAKE algorithm was used to constrain all bonds involving hydrogen. The temperature of each system increased gradually from 0 to 310 K over a period of 60 ps of NVT dynamics. This was followed by 200 ps of NPT equilibration at 310 K and 1 atm pressure. The resulting structures were then simulated for 100 ns. To determine the equilibrate state for trajectories sampling and convergence of simulations, root mean square deviation (RMSD) of all backbone atoms of the two simulated complexes (Figure S1, Supplementary Materials) were computed using initial structure as reference under the CPPTRAJ module [57]. Binding free energy of the complexes was accounted with Molecular Mechanics Generalized Born Surface Area (MMGBSA) and Molecular Mechanics Poisson Boltzmann Surface Area (MMPBSA) protocols under MMPBSA.py module [58] implemented in AMBER 14 using 1000 snapshots extracted from the last 5 ns of NPT-MD trajectories. 

### 4.3. Preparation of Gold Nanoparticle-DARPin Conjugates 

Gold nanoparticles (GNPs) used in the experiments were established by mixing 900 μL of GNPs solution and 100 μL of Ank^GAG^1D4 and Ank^GAG^1D4-Y56A at the concentration of 1000 μg/mL. The solution was incubated for 1 h in a shaking incubator. Next, 150 μL of bovine serum albumin (BSA) was added to the GNPs solution and incubated for an additional 1 h in a shaking incubator. The GNPs solution was centrifuged at 5000 rpm for 3 min and the pellet obtained was reconstituted in 1 mL of phosphate buffer saline (PBS) (pH 7) for measuring the optical density (OD) of GNPs. The recombinant CA was expressed in the baculovirus (BV) expression system [59,60] and purified by affinity chromatography on the HisTrap column, using ÄKTA Prime™ plus (GE Healthcare, Piscataway, NJ, USA). The protein concentration was quantified using a BCA Protein Assay from Pierce™ (Thermo Fisher Scientific, Waltham, MA, USA). 

### 4.4. Nanoparticle Tracking Analysis 

All NTA measurements were performed using 300 μL of the sample under NS300 Particle Measuring Instrument from NanoSight Ltd. (NanoSight, Worcestershire, UK). The size and distribution of AnkGAG1D4 and DARPin-conjugated GNPs was first characterized by diluting the samples in PBS to obtain optimal OD at 0.002. The binding activity of DARPin-conjugated GNPs and CA was measured by mixing diluted DARPin-conjugated GNPs with 100 μg/mL of purified HIV-1 CA protein in a 1:1 ratio. Particle movement was monitored for 60 s long by NTA to determine the size of DARPin-conjugated GNPs before and after interacting with CA. 

### 4.5. Validation of the Binding Activity Using Electrochemical Impedance Spectroscopy 

An Autolab PGSTAT204 (Metrohm, KM Utrecht, The Netherlands) and NOVA software were used in this study. A three-electrode system consists of indium tin oxide (ITO) as the working electrode, Ag/AgCl (3.0 M KCl) as the reference electrode and a platinum wire as the counter electrode was used. A clean ITO surface was modified with 5 mM of 1,4-phenylenediamine by CV technique at a scan rate of 100 mVs^−1^ for 2 cycles between +0.2 V and −0.6 V versus Ag/AgCl. 5 mM of the 1,4-phenylenediamine solution was prepared in 0.5 M HCl aqueous solution, to which 10 mM NaNO_2_ was added to generate the aryl diazonium cation. The diazonium cation solution was deaerated with nitrogen flow and allowed to react for at least 10 min prior to fabrication of the ITO surface. Next, the fabricated ITO surface was rinsed with Milli-QTM water and dried under a stream of nitrogen gas. Surface 1 (Figure 5) was incubated in an aqueous solution (60 μL), which contains 5 mM of NaNO_2_ and 0.5 M of HCl, for 15 min. After incubation, the ITO plate was rinsed with Milli-QTM water and dried under a stream of nitrogen gas. For Surface 2 (Figure 5), the fabricated ITO surface was incubated with 60 μL of Ank1D4 conjugated colloidal gold at room temperature (25 °C) for 3 h. Then the ITO plate was rinsed with Milli-QTM water and dried under a stream of nitrogen gas. Finally, the ITO surface (Surface 2 in Figure 5a) was used to detect CA protein (200 μg mL^−1^) by incubating the ITO plate with CA for 30 min. The detection of CA was monitored by electrochemical impedance spectroscopy (EIS) measurement performed with a DC potential of 0.2 V, a frequency range of 0.1–10,000 Hz and amplitude of 0.01 V. Surface characterization of ITO by CV technique in phosphate buffer solution (0.05 M of KCl and 0.05 M of K_2_HPO_4_/KH_2_PO_4_) containing 1 mM of [Fe(CN)6]^4−/3−^ at a scan rate of 100 mVs^−1^ for 2 cycles between +0.2 V and −0.6 V versus Ag/AgCl.

## Figures and Tables

**Table 1:** Binding affinity derived from MMGBSA and MMPBSA for Ank^GAG^1D4-CA and Ank^GAG^1D4-Y56A-CA complexes. EEL, MM electrostatic energy; vdW, van der Waals energy while the polar and non-polar term for MMGBSA/MMPBSA are EGB/EPB and ESURF/ENPOLAR, respectively.

Δ*E*_binding_ (kcal/mol)	Ank^GAG^1D4-CA	Ank^GAG^1D4-Y56A-CA
EEL	−118.70	−92.45
vdW	−219.39	−87.13
E_GB_	289.91	157.75
E_SURF_	−12.21	−9.23
Δ_GB_	−60.38	−31.06
E_PB_	280.89	129.06
ENPOLAR	−20.29	−16.55
Δ_PB_	−77.49	−67.07

## Data Availability

The data presented in this study are available within the article.

## Supplementary Materials

The following are available online, Figure S1: RMSD of Ank^GAG^1D4-CA and Ank^GAG^1D4-Y56A-CA complex throughout 100 ns simulations time; Table S1: Hydrogen bond pairs involving water molecules in Ank^GAG^1D4-CA and Ank^GAG^1D4-Y56A-CA complex.

## Sample Availability

Samples of the compounds are available upon request.
