# Supplementary material for: Protein-Protein Interactions: Insight from Molecular Dynamics Simulations and Nanoparticle Tracking Analysis
Source: Molecules. 2021 Sep 20;26(18):5696. doi: 10.3390/molecules26185696 (PMC8472368; doi:10.3390/molecules26185696)
Supplement: Supplementary file 1 [file molecules-26-05696-s001.zip › molecules-1373960-supplementary.pdf]

Supplementary Materials

Protein-Protein Interactions: Insight from Molecular Dynamics Simulations and Nanoparticle Tracking Analysis

Wei Lim Chong [1], Koollawat Chupradit [2,3], Sek Peng Chin [4], Mai Mai Khoo [1], Sook Mei Khor [1], Chatchai Tayapiwatana [2,3], Piyarat Nimmanpipug [5,6], Weeraya Thongkum [3,7] and Vannajan Sanghiran Lee [1,6,*]

[1] Department of Chemistry, Faculty of Science, Universiti Malaya, Kuala Lumpur 50603, Malaysia;
wlchongwilliam@gmail.com (W.L.C.); maimaikhoo1411@siswa.um.edu.my (M.M.K.);
naomikhor@um.edu.my (S.M.K.)
[2] Division of Clinical Immunology, Department of Medical Technology, Faculty of Associated Medical Sciences, Chiang Mai University, Chiang Mai 50200, Thailand;
kool_krub@msn.com (K.C.);
asimi002@hotmail.com (C.T.)
[3] Center of Biomolecular Therapy and Diagnostic, Faculty of Associated Medical Sciences, Chiang Mai University, Chiang Mai 50200, Thailand; weeraya.t@cmu.ac.th
[4] Department of Pharmaceutical Chemistry, Faculty of Pharmacy, Universiti Malaya, Kuala Lumpur 50603, Malaysia; spchin@um.edu.my
[5] Department of Chemistry, Faculty of Science, Chiang Mai University, Chiang Mai 50200, Thailand; piyarat.n@cmu.ac.th
[6] Center of Excellence for Innovation in Analytical Science and Technology (I-ANALY-S-T), Chiang Mai University, Chiang Mai 50200, Thailand
[7] Center of Innovative Immunodiagnostic Development, Department of Medical Technology, Faculty of Associated Medical Sciences, Chiang Mai University, Chiang Mai 50200, Thailand
* Correspondence: vannajan@um.edu.my

Figure S1

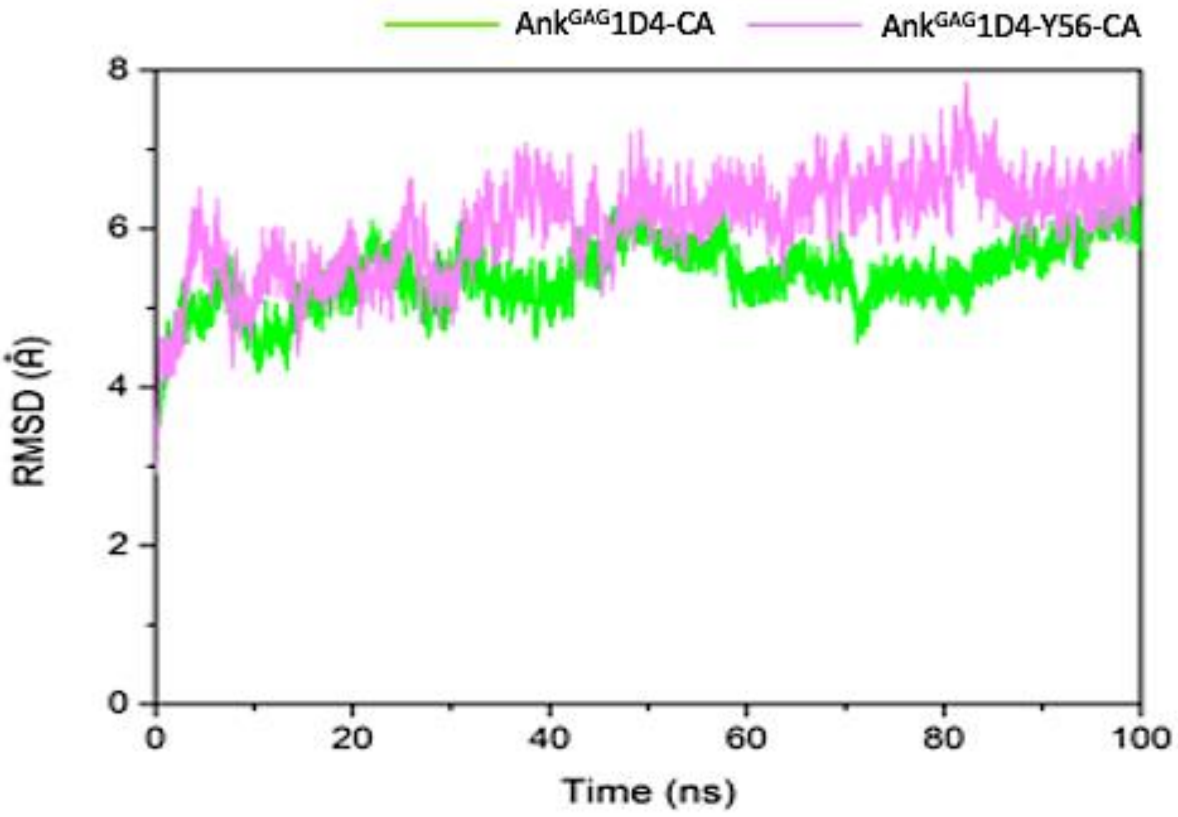

Figure S1. RMSD of Ank^{GAG}1D4-CA and Ank^{GAG}1D4-Y56A-CA complex throughout 100 ns simulations

Table S1

Table S1. Hydrogen bond pairs involved water molecules in Ank^{GAG}1D4-CA and Ank^{GAG}1D4-Y56A-CA complex

Hydrogen bonding pair in Ank^{GAG}1D4-CA		Fraction (%)	Hydrogen bonding pair in Ank^{GAG}1D4-Y56A-CA		Fraction (%)
**(**P) I_1130@O	(A) Y_56@OH	79	(A) D_77@OD1	WAT_2245@O	67
(A) V_73@O	WAT_5964@O	58	(A) V_73@O	WAT_7336@O	57
(A) D_77@OD1	WAT_13750@O	56	(A) S_48@OG	WAT_2245@O	57
(A) S_48@OG	WAT_13750@O	51	WAT_1663@O	(A) T_49@OG1	85
WAT_7557@O	(A) Y_56@OH	92	WAT_2245@O	(A) Y_86@OH	79
WAT_14761@O	(A) T_49@OG1	73	WAT_2245@O	(A) H_52@ND1	76
WAT_13750@O	(A) Y_86@OH	72	WAT_12568@O	(A) R_77@NH1	75
WAT_12460@O	(A) T_115@OG1	54	WAT_6435@O	(A) L_117@N	55
WAT_8890@O	(A) L_117@N	51	WAT_13894@O	(A) T_115@N	54

Only hydrogen bonding pairs that remained for more than 50% of the simulations time are shown. (A) denotes residues Ank^{GAG}1D4/ Ank^{GAG}1D4-Y56A while (P) denotes residues CA.
